# Chinese expert consensus on the treatment of MMD

**DOI:** 10.1186/s41016-023-00318-3

**Published:** 2023-02-23

**Authors:** Xiang-Yang Bao, Lian Duan

**Affiliations:** Department of Neurosurgery, the First Medical Centre, Chinese PLA General Hospital, 8 Dong-Da Street, Fengtai District, Beijing, 100071 China

## Abstract

Moyamoya disease (MMD), also known as spontaneous occlusion of the circle of Willis, is defined by progressive stenosis or occlusion of the internal carotid arteries, and it can progress to the anterior, middle, and posterior cerebral arteries. As these arteries are gradually stenosed, a collateral network of capillaries develops at the base of the brain, producing the characteristic reticulate appearance (“puff of smoke”) on angiography. Therefore, it was named by Suzuki and Takaku in 1969 after the Japanese term “moyamoya” (Suzuki and Takaku, Arch Neurol 20:288–299, 1969). MMD is most common in East Asian countries such as Japan and Korea, and it shows a slight female predominance. MMD is mainly characterized by ischemia and hemorrhage. Hemorrhagic MMD is very rare in children, and most cases occur in adults due to the rupture of the compensatory blood vessels, which often leads to hemorrhagic symptoms (Scott and Smith, N Engl J Med 360:1226–1237, 2009). In recent years, the diagnosis rate has increased with the popularization of imaging techniques. However, the pathogenesis of MMD is still not completely understood, and there is currently no evidence to suggest that drug treatment can delay or even reverse the progression of MMD. The current drug treatment for in MMD only targets its clinical symptoms, including ischemia and hemorrhage. The main choice of treatment for MMD is surgical revascularization. As an increasing number of hospitals have developed surgical treatment for MMD, our compiling group has jointly discussed the formulation of a consensus among Chinese experts on the treatment of MMD.

Since the cause of MMD is largely unknown, medical treatment is still insufficient, and there are no effective drugs for treatment; treatment mainly involves treating ischemic and hemorrhagic symptoms. The treatment of MMD is mainly based on intracranial and extracranial revascularization surgery, and an increasing number of hospitals in China are performing surgical treatment for MMD. However, there is still a lack of standards of diagnosis and treatment. In view of this, we jointly discussed the formation of consensus among Chinese experts on the treatment of MMD. Uniform treatment standards are still lacking.

## Conservative treatment

There is currently no drug that can effectively control or reverse the pathogenesis of MMD. As such, symptomatic supportive treatment and perioperative treatment are the most common types of treatment. Clinically, simple medical therapy is applied to patients with MMD who have no surgical indications (slight symptoms or intolerable surgery), the main goal of which is to prevent cerebral thrombosis, maintain sufficient cerebral blood volume, and target the symptoms of the disease, including headache and epilepsy.

### Antiplatelet drugs

Internationally, there is no consensus about whether anti-platelet drugs should be used in patients with MMD. Although it is generally believed that the ischemic events in patients with MMD are caused by severe hypoperfusion, several studies have found microemboli in the blood circulation of patients with MMD, suggesting that arterial-to-arterial embolism is also involved in ischemic events [1]. Some scholars believe that taking aspirin is very useful for the treatment of MMD, especially after the patient undergoes revascularization, because they believe aspirin can prevent small blood clots and maintain blood flow in the reconstructed blood vessels [2–4]. However, some studies have pointed out that aspirin cannot improve the symptoms of focal cerebral ischemia and may increase the incidence of cerebral hemorrhage [5]. Therefore, the current use of aspirin for MMD is only performed empirically. For patients with ischemic MMD, whether surgery or not, oral aspirin is recommended in the 2012 Japanese MMD guide [6]. Aspirin is not recommended for patients with asymptomatic MMD. However, a multi-center survey data from Japan in 2018 showed that taking aspirin before admission can improve neurological function in patients with non-hemorrhagic MMD [7]. Moreover, studies have shown that taking aspirin can effectively reduce the incidence of recurrent stroke, improve the survival rate of patients without stroke, and increase the risk of cerebral hemorrhage postoperatively in patients with MMD [8].

Based on this previous research, we recommend that antiplatelet drugs should be given to patients with chronic ischemic and acute cerebral infarction. If the patient is intolerant to aspirin or if it is ineffective in preventing ischemic attacks, other drugs such as clopidogrel can be used instead. However, as long-term combined use of aspirin and clopidogrel has been shown to increase the risk of bleeding, the combination is not recommended. Moreover, for patients with asymptomatic and hemorrhagic MMD, the risk of rebleeding may increase, and aspirin should be used with caution, but for patients with high-risk factors such as arteriosclerosis (e.g., smoking, hypertension, diabetes, hyperlipidemia). Hemorrhagic patients should generally start aspirin 3–6 months after the start of bleeding.

### Calcium ion antagonists

Some calcium antagonists such as nimodipine and nicardipine can selectively dilate cerebral blood vessels. Previous studies have reported that calcium antagonists can improve postoperative clinical function and reduce stroke events in children with ischemic MMD [8]. However, when the dose is too high, they may cause hypotension, reduce cerebral perfusion, and aggravate the ischemia and hypoxia associated with MMD. We also found that early application of these drugs may cause the clinical ischemic symptoms to worsen. This may be related to individual differences in vascular pathology in patients with MMD, which can impact the patients’ sensitivity to the drug. Therefore, we do not recommend such drugs for the treatment of MMD.

In patients with MMD and hypertension, calcium antagonists such as nifedipine and amlodipine can be used as antihypertensive drugs to maintain blood pressure. For patients with MMD with paroxysmal severe headache, especially migraine and dizziness, as a clinical symptom, calcium ion antagonists such as flunarizine hydrochloride can be used for symptomatic treatment, but the patient’s blood pressure must be monitored during the treatment. In cases where hypotension is detected, the drug may be discontinued or the dose adjusted. In addition, calcium ion antagonists can be used for subarachnoid hemorrhage in patients with MMD. Nimodipine injection is commonly used to prevent brain damage caused by cerebral vasospasm. It is also necessary to closely monitor blood pressure changes during use.

### Statins

Statins can mobilize bone marrow endothelial progenitor cells, induce endogenous cell proliferation, enhance neuronal plasticity, promote angiogenesis, and increase local tissue blood supply [9]. Several domestic studies have shown that simvastatin combined with revascularization surgery can significantly improve blood supply and promote angiogenesis in cerebral ischemic areas, and establish collateral circulation [10]. We have also observed in clinical practice that the application of statins can stabilize vascular endothelial function and improve the postoperative neurological function and vascular remodeling effect with no obvious side effects. Despite these advantages, the widespread use of statins in MMD currently lacks objective evidence.

### Neuroprotective drugs

The most commonly used neuroprotective drugs are edaravone, butylphthalide, oxiracetam, and some traditional Chinese medicine preparations (such as ginkgo preparations [Ginaton, Salvia]), which are used to improve microcirculation. Edaravone is a brain-protective agent that scavenges free radicals and inhibits lipid peroxidation, thereby inhibiting oxidative damage of brain cells, vascular endothelial cells, and nerve cells. Clinical studies have shown that edaravone can prevent brain edema and cerebral infarction progression and alleviate the accompanying neurological symptoms to inhibit delayed neuronal death. Studies in Japan have shown that perioperative edaravone can reduce the neurological deficits associated with hyperperfusion after revascularization in adult moyamoya patients [11].

Butylphthalide is a synthetic racemic n-butylphthalide. Clinical studies have shown that butylphthalide can improve the damage to central nervous function in patients with acute ischemic stroke, thereby improving the neurological deficit in patients. Although butylphthalide can be used in the treatment of patients with MMD, there is currently no objective clinical evidence for its efficacy. Indeed, only one study has shown that postoperative administration of butylphthalide can alleviate the neurological deficit during perioperative period after revascularization in patients with MMD [12]. However, the use of this drug in patients with MMD still needs to be undertaken cautiously, and further study is required to confirm its efficacy.

Some promoting microcirculatory traditional Chinese medicine, because of their exact effect in improving microcirculation, including Ginaton and Salvia miltiorrhiza, can be used in the ischemic attack and perioperative period of MMD to relieve symptoms and prevent perioperative ischemic events. In addition to drugs, patients with acute neurological deficits as a result of acute stroke should undergo rehabilitation as soon as possible; this may involve Chinese medicine acupuncture, massage, and device-assisted treatment. Recently, the application of remote ischemic conditioning (RIC) in the treatment of MMD has been reported. RIC is a training method that causes multiple local ischemia and reperfusion of the limb by repeated and brief compression and release of limbs and other organs. RIC has been shown to improve cerebral blood perfusion in patients with MMD, thereby reducing the recurrence rate of ischemic stroke and reducing the frequency of transient ischemic attack (TIA) attacks. RIC can also improve the effect of revascularization on cerebral blood perfusion and reduce surgical complications [13]. However, the use of RIC has only been reported in a few clinical trials and is not widely carried out in the treatment of MMD; therefore, its role and efficacy in treatment need to be further verified.

## Surgical treatment

Surgery is currently the most effective treatment for MMD and can be divided into direct revascularization, indirect revascularization, and combined revascularization (direct + indirect). The purpose of surgery is to use the external carotid artery system either directly or indirectly to increase intracranial blood flow, thereby improving cerebral blood flow and cerebral blood flow reserve. At present, the idea that revascularization can effectively increase cerebral blood flow and reduce the incidence of ischemic stroke has been accepted by most scholars, but its effectiveness in preventing bleeding still requires long-term follow-up study.

### Surgical indication

The main surgical indications for MMD are as follows: (1) symptoms of cerebral ischemia associated with disease, including TIA, rind, cerebral infarction, cognitive decline, involuntary limb movement, headache, and seizures; (2) evidence of decreased cerebral blood flow reserve capacity, including local cerebral blood flow (CBF) and cerebrovascular reserve capacity (CVRC) reduction; (3) cerebral hemorrhage associated with disease, excluding other causes; and (4) exclusion of other surgical contraindications.

The surgical indications for patients with hemorrhagic MMD are inconsistent. Following vascular angiography, there is evidence that cerebral angiography in patients with MMD shows a decrease in smog-like blood vessels or the disappearance of aneurysms associated with hemodynamic changes. Furthermore, revascularization reduces the hemodynamic load of the collateral vessels [14, 15]. It has also been found that patients with hemorrhagic MMD have a significantly lower rate of rebleeding after surgery [16–18] compared to patients who undergo conservative treatment. Moreover, many studies have reported that revascularization can prevent rebleeding [19–21]. Indeed, a multicenter prospective randomized controlled clinical trial in Japan in 2014 showed that cerebral revascularization can reduce the rate of rebleeding from 31.6 to 11.9%; this study is currently the strongest evidence supporting surgery for hemorrhagic MMD [22]. It is currently believed that the initial cause of bleeding in hemorrhagic MMD is a reduction in cerebral blood flow. Hemorrhage is caused by over-compensation for cerebral ischemia. In theory, improving intracranial ischemia can reduce the compensatory pressure and reduce the risk of rebleeding. Most experts in China believe that surgery for hemorrhagic MMD can increase cerebral blood perfusion and reduce the rate of rebleeding. Therefore, we recommend that while patients with hemorrhagic MMD have the option of active surgical treatment, there must be strong communication between physicians and patients and their families to keep them fully informed.

The surgical indications for children with MMD should be relaxed when appropriate because MMD in children progresses faster than in adults. In line with this, 3/4 of children with MMD have a lower learning ability than children of the same age 4 years after onset; thus, early diagnosis and active intervention before irreversible brain injury occurs are important for children with MMD to obtain good clinical prognosis [23, 24]. Indeed, early treatment can restore more than 90% of children to a normal life, and even learning ability is significantly improved compared with that preoperatively. Asymptomatic MMD cases with accidental discovery can be observed conservatively if there is no hemodynamic damage, but must be followed closely. When clinical symptoms or hemodynamic changes are found, surgery can be considered.

### Timing of surgery

In principle, it is recommended that the diagnosis be confirmed as soon as possible after surgery. However, in some cases, the time of surgery should be postponed. For example, in patients with acute or subacute cerebral infarction on the diffuse image of the head MRI, immediate surgical treatment may increase the risk of perioperative stroke. Therefore, it is recommended that this patient group be conservatively treated and observed several weeks later to determine the patient’s own recovery status; surgical revascularization may be considered after approximately 1–3 months. In addition, we do not recommend immediate surgery for patients with recent frequent TIA as this indicates that the patient has unstable hemodynamics and it is prone to cerebral infarction during the perioperative period. Therefore, we recommend that these patients undergo surgery after conservative treatment. In the acute phase of cerebral hemorrhage, conservative treatment or surgical removal of the hematoma should be performed depending on the size and location of the intracranial hematoma. The removal of the hematoma should be carried out under forced conditions, in which the superficial temporal artery should be preserved for secondary revascularization. After the condition has stabilized, the hematoma becomes completely absorbed, and elective revascularization may be considered; this is generally 1–3 months after the removal of the hematoma.

### Surgical methods

#### Direct revascularization

In most cases, direct revascularization uses the superficial temporal artery as the donor artery and sometimes uses the deep iliac artery or occipital artery. The recipient artery usually chooses the cortical branch of the middle cerebral artery. The classical procedure is superficial temporal artery-middle cerebral artery (STA-MCA) anastomosis. In addition, the superficial temporal artery-anterior cerebral artery branch or the occipital artery-the posterior cerebral artery could be chosen according to the patient’s intracranial hypoperfusion area.

The main advantage of direct revascularization is that matching of the extracranial artery with the cortical branch of the intracranial artery can immediately increase the blood flow of the ischemic brain and rapidly improve the hemodynamic state. However, direct blood supply reconstruction is difficult and requires rigorous training by the surgeon. In addition, the procedure requires the patient to have a good vascular condition because in the late stages of MMD, or in young children, the diameter of the tube is small and the vessel wall is more fragile, making the anastomosis difficult to perform. Due to the complicated technical process, the operation time is long, and the cortical arteries need to be temporarily clamped during the operation, resulting in a higher incidence of perioperative ischemic complications. In addition, postoperative hyperperfusion syndrome is a known complication after direct revascularization that causes neurological deterioration in patients [25, 26].

#### Indirect revascularization

The basic principle of indirect revascularization is to cover the surface of the ischemic brain with blood vessels or various connective tissues derived from the external carotid artery system. The surgical methods are classified according to the different tissues used, mainly including brain-dural tape placement: encephalo-duro-synangiosis (EDS), encephalo-myo-synangiosis (EMS), encephalo-duro-arterio-synangiosis (EDAS), brain-hard encephalo-duro-arterio-myo-synangiosis (EDAMS), encephalo-galeo-synangiosis (ESS), brain-cranioplasty, encephalo-periosteal-synangiosis (EPS), and multiple bur holes (MBH). The most common clinical methods are EDAS and EDAMS; some surgical procedures cannot be performed due to limited efficacy and the potential for serious complications.

In contrast to the direct technique, temporary blockage of the middle cerebral artery branch is not required during indirect revascularization. Moreover, the surgery is relatively simple, and it is easier to widely spread and apply in China. Indirect revascularization also has a shorter operation and hospitalization time, less trauma, and a low economic burden, and it is more frequently used for children and adult patients with complicated conditions. Furthermore, indirect revascularization avoids brain tissue ischemia caused by factors such as temporary blockage of cortical blood vessels and excessive anesthesia and also avoids hyperperfusion syndrome caused by a sudden increase in blood flow caused by complications of direct anastomosis. However, due to the establishment and function of collateral circulation, it usually takes more than a week for the cerebral blood flow of the patient to improve, and some patients may have cerebral ischemic events during this period.

It is worth noting that, compared to direct revascularization, indirect reconstruction is mild and sustained with regard to improving the blood supply in the ischemic areas of the brain. Some studies have reported that the blood supply of direct revascularization has gradually degraded or even disappeared in patients during long-term review. Instead, the blood supply is formed by indirect reconstruction, suggesting that blood supply from direct revascularization can only play a short-term role, while the blood supply formed by indirect reconstruction is persistent and stable. In addition, the blood supply formed by indirect reconstruction is based on the degree of ischemia of the brain tissue, which can naturally and reasonably coordinate and distribute blood supply in different areas. However, direct reconstruction involves the artificial selection of receptors. It is difficult to choose accurately and reasonably, which will result in over-perfusion in some areas and under-perfusion in other parts. The time required to form a new collateral circulation after indirect revascularization is generally considered to be in the region of weeks or even several months. However, a more recent view is that the postoperative neonatal collateral circulation takes only a few days to form, which is consistent with our view [27]. Indeed, we reported that newborn collateral circulation to the blood supply to the ischemic brain tissue could be observed in a patient only 1 week after indirect reconstruction [28].

#### Combined revascularization

In summary, direct revascularization and indirect revascularization have unique advantages and disadvantages. If a surgical procedure is adopted, direct surgery and indirect surgery are included, and their respective advantages are exerted; then, in theory, the effect will be better, hence the introduction of combined reconstruction surgery. Commonly used joint reconstruction methods include superficial temporal artery-brain artery anastomosis combined with cerebral-muscle musculature (EMS), brain-dural-arterial (EDAS), brain-dural-arterial-muscle occlusion (EDAMS), brain-dural-diaphragm-arterial-periosteal occlusion (EDMAPS), and brain-dural-cap-like aponeurosis (EDGS).

#### Perioperative management

During the perioperative period, it is necessary to maintain blood pressure, ensure a normal blood carbon level, maintain proper fluid balance, and pay attention to ischemic complications (including the non-surgical side). Neurological symptoms may occur in the acute phase after revascularization, and some clinical conditions, such as brain hyperperfusion syndrome, should be considered when performing hemodynamic assessment. Oral antiplatelet drugs are recommended during the perioperative period to prevent ischemic events, although they may cause some difficulties in intraoperative hemostasis; they may also reduce the incidence of postoperative ischemic events.

#### Choice of surgical method

With regard to direct, indirect, and combined revascularization, there have been no large, multicenter, prospective, randomized controlled trials that have validated which surgical approach is most effective. The current literature on surgical comparisons is a retrospective analysis. A systematic review of 35 published articles on surgical outcomes showed no significant difference in postoperative stroke rates among the three surgical groups, but did find that direct revascularization was superior to indirect revascularization in preventing recurrent stroke [29]. Another study reviewed 47 cases of 2013 adult patients with MMD and showed that the prognosis of the direct surgery group was higher than that of the indirect surgery group. Both studies seem to agree that direct or combined revascularization are superior to indirect revascularization [30] in adults with MMD. However, another retrospective analysis of 4197 patients with MMD in 33 studies (5 in children) showed no significant difference in the surgical outcome between indirect and combined revascularization during the 10-year follow-up period, but both were superior to direct revascularization; in the 4-year follow-up period of adult patients, indirect surgery is superior to direct surgery [31]. Therefore, it is believed that both indirect and combined revascularization have advantages in long-term outcomes in both children and adults with MMD. In addition, international studies have compared joint reconstruction; the indirect effect of reconstruction surgery on adults with non-hemorrhagic MMD showed that although patients who underwent combined surgery had more frequent perioperative complications, there was no significant difference in clinical prognosis. Therefore, it was considered that indirect revascularization could become a reliable alternative to combined surgery [32].

In summary, most scholars believe that direct or combined surgery is better than indirect surgery early, especially for adult patients. However, this view has also changed in recent years, and some scholars now believe the long-term effect on patients is the same, regardless of whether they undergo direct/combined surgery or indirect surgery, since there are no essential differences between the surgical methods. Focus should be on individualized treatments. Multicenter and prospective randomized controlled trials have been conducted to compare the effects of different surgical treatments for MMD. However, due to some objective conditions, there will be some difficulties and deviations in the trial. We also expect the trial to have a conclusion to end the current controversy. Before the final conclusion determined, we recommend that each moyamoya treatment center combine its own experience and advantages to select the most appropriate surgical method for individualized treatment without excessive entanglement in the choice of surgical approach. With regard to the treatment of MMD, the focus should be on perioperative management and prevention of postoperative complications, both of which can improve long-term neurological function and benefit patients.

Declaring expert consensus is stage recognition, based on the current literature, combined with the personal experience of the listed experts, still needs to be improved with the advancement of diagnosis and treatment technology. This expert consensus is only for reference by clinicians in the process of diagnosis and treatment. It does not have legal effects and does not exclude individualized successful diagnosis and treatment experience that have not been included in the consensus.

## Data Availability

Data sharing is not applicable to this article as no datasets were generated or analyzed during the current study.
